# Effectiveness of health education intervention on diabetes mellitus among the teachers working in public sector schools of Pakistan

**DOI:** 10.1186/s12902-022-01110-7

**Published:** 2022-07-30

**Authors:** Ramesh Kumar, Sidra Rehman, Gul Muhammad Baloch, Muskan Vankwani, Ratana Somrongthong, Sathirakorn Pongpanich

**Affiliations:** 1 Department of Public Health, Health Services Academy, Islamabad, Pakistan; 2 College of Public Health Sciences, Chulalongkorn University, Bangkok, Thailand; 3 Dow International Medical College, Karachi, Pakistan

**Keywords:** Health education, Diabetes, Government schools, Prevention, Teachers, Communicable diseases

## Abstract

**Background:**

Diabetes Mellitus (DM) is considered as one of the major public health problems globally. Health education strategies can help in managing blood glucose level and complications among DM patients. Health education intervention is effective to manage and control the blood glucose levels among diabetic patients. This study explored the effectiveness of health education intervention on DM among school teachers in public sector schools of Pakistan.

**Methods:**

This was quasi-experimental study where baseline & end line assessments were conducted on teachers of public sector schools of Sindh province, Pakistan, from October to December 2019. Pretested structured questionnaire was used in this study. Participants (*n* = 136). were randomly selected from the list of government schools registered with district education department An intervention comprised of health education sessions with DM patients was undertaken after conducting baseline assessment followed by end line assessment. The institutional review board of Health Services Academy Pakistan ethically approved this study.

**Results:**

All the respondents completed post-test with mean ± SD age of participants being 39.2 ± 1.34 years. Female teachers comprised 65% out of which 70% were living in rural areas. Knowledge on DM pre-test score was 20.03 ± 3.31 that increased in post-test to 49.11 ± 2.21 (*p* < 0.05). Mean score of information on symptoms and causes of DM was 1.98 ± 0.21 for pre-test whereas for post-test it was 4.78 ± 0.12 (*p* < 0.05). The effect of intervention was significant on diabetes related complications (*p* < 0.05), symptoms (*p* < 0.05), overall score (*p* < 0.05) and preventive practices (*p* < 0.05).

**Conclusions:**

The study provides evidence of the importance and effectiveness of health education intervention related to diabetes among school teachers, which has a positive impact on the knowledge and practices. We concluded that the health education session sensitized the teachers and they can bring cogent changes to enhance their knowledge about diabetes and its risks.

## Background

Diabetes Mellitus (DM) is the chronic non-communicable disease affecting 425 million people around the globe and is considered as one of the major public health problems [1]. South Asia added more than 72 million people with diabetes and Pakistan contributes 15% prevalence of diabetes among general public [2–5]. DM type-2 is more common among South Asian population and these individuals are at high risk of diabetes-related complications [2]. Hence diabetes is considered as serious condition that can be prevented through proper information and provision of proper health education interventions [6]. Provision of health education sessions produces incredible change in the knowledge, attitude, and the behaviour among the patients to manage their self blood sugar level more efficiently [7]. DM type 2 is a common multifactorial genetic syndrome that affects the individual’s families. Different risk factors like unhealthy life style, smoking, alcohol consumptions, family history and diet are the major leading cause of type 2 diabetes; uncontrolled blood sugar may lead to further complications of renal failure, myocardial infarction, amputation, blindness, and hypertension [8, 9].

Proper compliance with dietary guideline, self-care, physical activity, and treatment may reduce the progression of disease [1, 10]. The most common cause of increased prevalence of diabetes among the general population is lack of knowledge and physical inactivity [11]. Health care providers strongly recommend the diabetic patients to exercise regularly, to follow their dietary plan and to adhere with medication schedule [12]. Moreover, it is important for diabetic patients to have a regular health check ups for their personal health [13]. Health education strategy is very important in maintaining of blood sugar level, which will help population to be aware of the disease and its consequences [14]. Health care facilities should provide necessary information about diabetes on regular patient counselling sessions along with their routine treatment that might benefit their health outcome positively [15]. Health education trainings /workshops/ seminars with proper information are the utmost need of the patients for timely reduction of morbidity and enhance the confidence among diabetic patients by self-care diabetes management [16].

Public Schools are the common institutions, where the students of various backgrounds interact with teachers. Schools are main source for dissemination of health information and knowledge on disease management in the community. Hence, the school health education is found to be very imperative to adopt healthy behaviours for prevention against non-communicable diseases [17, 18]. Moreover, school-based health interventions have been proved to enhance the knowledge on management of type 1 diabetes among the school going children [19]. Hitherto, there is scarcity of published evidence to support the effectiveness of health education intervention for diabetes among school teachers in Pakistan. The objectives of this study were to assess the effectiveness of health education intervention on diabetes among teaching staff working in public schools of Sindh. Teachers are the backbone of society to teach and train our new generation in public schools and they should be well aware on diabetes. Therefore, this study was conducted to improve the knowledge about diabetes among school teachers working in public schools of Sindh province of Pakistan. This study has also proved good evidence to incorporate this health education information in the regular academic curricula on diabetes.

## Methods

### Study design, respondents and site

This was quasi-experimental study with baseline assessment followed by the intervention and end line assessment on the teachers working in public sector/ government schools of district Sukkur Sindh from October–December 2019. In total 136 respondents were randomly selected from the secondary schools and participated in baseline and end line assessments. The respondents completed the questionnaire at baseline, participated in the designed health education intervention for DM prevention and involved in end line assessment to compare the difference.

### Study instrument

The Health Literacy Survey in Europe HLS-EU-Q47 which is a structured, piloted and pretested tool previously used in six Asian countries was used in this study [20]. The experts checked the validity of this instrument. This tool comprised the questions related to awareness level, education, and knowledge about DM and guidance on preventive measures.

### Statistical analysis

Descriptive statistics included frequency, percentages and knowledge level of the respondents, which was analysed by using statistical package for the social science (SPSS) version 24. All the questions on knowledge, preventive practices, and health literacy information related to DM were scored from lower to higher level as per the responses. Mean scoring of baseline and end line assessments was compared and paired t-test was used to calculate the *p* values for statistical significance.

### Intervention

Health education session for DM prevention continued for 100 minutes and participants and facilitators were oriented during these sessions to share the information through direct face-to-face presentation and sharing information and communication materials. Researchers developed four modules for quality health education session, which were based on intensive literature and an information-motivation behavioural skills (IMB) model [6, 14, 15]. This session was followed by a separate module and presented the relevant information on DM among the participants. These modules covered the topics of the earlier sessions on diabetes risk factors, causes, epidemiology, diagnosis, blood sugar level, its complications, treatment, and preventive practices about knowing their normal blood sugar level, use of sugar in diet, physical activity and its frequency, smoking habit and information seeking behaviour. Pre-test was done prior to this intervention and post-test data were collected immediately after the intervention.

## Results

All 136 respondents completed the intervention with response rate of (100%). Socio-demographic characteristics of the participants are presented in Table 1.

**Table 1:** Socio-demographics characteristics of the respondents

Variables	Categories	Frequencies (n)	Percentage (%)
**Sex**	Male	48	35.3
	Female	88	64.7
**Age (years)**	18–25	0	0
	26–33	23	16.9
	34–41	53	39.0
	42–49 Years	41	30.1
	50 Years and above	19	14.0
**Qualification**	Bachelor	50	36.8
	Masters	47	34.6
	MS / M. Phil	39	28.7
**Experience**	1–5 Years	17	12.5
	6–10 Years	12	8.8
	11–16 Years	35	25.7
	17 years and above	72	53.0
**Monthly income**	< 200 US $	23	16.9
	200–500 US $	72	52.9
	> 500 US $	41	30.2
**Source of information on diabetes**	Media	72	52.9
	Peers and friends	17	12.5
	Books news and conferences	19	14.1
	Health Professionals	28	20.5

The mean ± SD age of respondents was 39.2 ± 1.34 years. 65% teachers were females, out of which 70% belonged to rural areas. Majority of the respondents were graduates and postgraduates and only 29% were Master of philosophy. Their monthly average income was 200–300 US$, with an average experience of 15 years in teaching. More than half of the participants got information on diabetes from media followed by health professionals, books, and peers/friends. T-test is parametric test that was used to check the underlying distribution of the variable of interest is normally distributed in this data. The health education intervention had a positive and statistically significant effect (*p* = < 0.05) on teachers’ knowledge, information and practices as presented in Table 2.

**Table 2:** Respondent’s Knowledge on diabetes Mellitus

Variables	Baseline-test (%)	Endline-test (%)	***p*** value
*Health Knowledge on diabetes*			
Symptoms	43	100	< 0.05
Treatment	8	100	
Types	8	100	
Health seeking advice from doctors	10	100	
Diabetes affects on other body organs	40	100	
Causes dehydration	30	100	
Eating different types of food can cause diabetes	12	100	
Physical regular exercise controls the diabetes	5	100	
*Information about symptoms and causes of diabetes*			
Genetic disease	2	100	< 0.05
Obesity	30	100	
Sedentary life style	22	45	
Stress	11	79	
Increased eating and drinking	33	100	
Headache and weakness	13	100	
Generalized weakness	19	100	
*Health Practices related variables*			
Check blood sugar level	2	30	< 0.05
Consultation with Doctor	10	35	
Interested to know about diabetes	10	100	
Diet plan can prevent diabetes	2	20	
Insulin is the treatment in type 2 diabetes	10	30	
Regular Exercise	8	80	
Restrict high sugar diet	10	100	

Overall mean score showed significant difference between baseline and end line assessments. The baseline mean score was20 ± 3.3 which increased to 49 ± 3.9 in post-test; the average difference and intervention effect was 29 among the respondents. Further, it was observed that all the variables scoresimproved after the intervention (Table 3). All the respondents completed post-test assessment that was done immediately after the intervention (direct session of 100 minutes).

**Table 3:** Overall significant change in mean knowledge of participants after the intervention

Section	Baseline-test	Endline-test	Absolute change	***p*** value
Diabetes and complication	1.989	4.784	2.795	< 0.05
Symptoms of diabetes	2.911	4.031	1.120	< 0.05
Preventive Practices	1.780	3.571	1.791	< 0.05
Total score (HE of diabetes)	20.03	49.11	29.08	< 0.05

## Discussion

Health education is an important intervention in preventing the general masses from non-communicable diseases. The respondent’s knowledge improved significantly after face-to-face 100 minutes’ session on diabetes. Research suggests that the timely information could prevent a large number of diabetes-related risk factors among the patients. Hence, this study has highlighted the value of health education sessions among teachers in rural areas where access to health facilities is limited [18]. Health Education intervention has proved to be effective for the prevention of diabetes among teaching staff. However, prior to this intervention, participants had limited knowledge about diabetes, which statistically improved after giving the health education sessions at their work place. These findings were supported by the interventional studies conducted in similar settings, where it has been proved that the health education can change individual’s knowledge and behaviours to prevent certain diseases and infections [1, 18, 21, 22]. Moreover, literature has also revealed the similar findings that the health literacy is an important strategy used for the better prevention against non-communicable diseases in the community [6, 7, 11–15]. Evidence suggests that the school-based interventions against preventable diseases have been proved as the best approach to educate students [19].

Health education level varies with different factors like education, experience, and income, as reported by these studies [4, 14]. Our study found that the teaching staff was not aware about diabetes initially, and that their knowledge after the health education intervention considerably increased. They were briefed on symptoms, treatments, types of the disease, and other important information related to the health and diabetes. The similar type of studies shows the significant improvement in the knowledge of population after the health education intervention [12, 14, 15, 19]. A previous study proved that the health education is associated with various factors such as sex, educational status, and place of care, which might influence knowledge, and self-management of diabetes, and glycaemic control in a Pakistani diabetic population [22]. The attitude and practices of the respondents changed a lot after session of health education on diabetes, life style and treatment [23]. The same results were obtained in the population where the intervention contained colour booklets, animated videos, face-to-face lectures, and practical display of eating platter. The results are similar with our study as health education intervention helps in managing the blood sugar and cholesterol level among the diabetic patients [11].

## Conclusion

Initially, before the intervention, the school teachers were unaware about the diabetes. Study concluded that the health education intervention, which included sessions at their workplace, had positively improved the knowledge of the respondents on diabetes. The participants were also made aware on the importance of dietary habits that helped increase their physical activity and its resultant benefits among diabetic patients. Time constraint was the major limitation in this study and the impact of sustained health education intervention on diabetes among the participants was not assessed. However, it is highly recommended for future studies to measure a change in knowledge and retention over time and further measure its impact on encouraging healthy behaviours that would minimise risk for the development of type II diabetes.

## Data Availability

The datasets used and/or analysed during the current study are available from the corresponding author on reasonable request.

## Abbreviations

HE: Health education
DM: Diabetes Mellitus
HLS-EU: Health literacy survey in Europe
